# Hypercholesterolemia Negatively Regulates P2X7-Induced Cellular Function in CD4^+^ and CD8^+^ T-Cell Subsets from B6 Mice Fed a High-Fat Diet

**DOI:** 10.3390/ijms23126730

**Published:** 2022-06-16

**Authors:** Tom Hutteau-Hamel, Amine Mellouk, Nicolas Trainel, Anne-Marie Cassard, Pierre Bobé

**Affiliations:** Institut National de la Santé et de la Recherche Médicale—INSERM, Université Paris-Saclay, UMR 996, 92140 Clamart, France; tom.hutteau@universite-paris-saclay.fr (T.H.-H.); amine.mellouk@universite-paris-saclay.fr (A.M.); nicolas.trainel@universite-paris-saclay.fr (N.T.); cassard.doulcier@universite-paris-saclay.fr (A.-M.C.)

**Keywords:** cholesterol, high fat diet, P2X7 receptor, T-cell subsets

## Abstract

We have previously showed that plasma membrane cholesterol and GM1 ganglioside content are responsible for the opposite sensitivity of mouse leukemic T cells to ATP. We also reported that the sensitivity of CD4^+^ and CD8^+^ T cells to ATP depends on their stage of differentiation. Here, we show that CD4^+^ and CD8^+^ T cells from B6 mice express different levels of membrane GM1 and P2X7 but similar levels of cholesterol. Thus, in CD4^+^ T cells, membrane cholesterol content negatively correlated with ATP/P2X7-induced CD62L shedding but positively correlated with pore formation, phosphatidylserine externalization, and cell death. By contrast, in CD8^+^ T cells, cholesterol, GM1, and P2X7 levels negatively correlated with all these ATP/P2X7-induced cellular responses. The relationship between cholesterol and P2X7-induced cellular responses was confirmed by modulating cholesterol levels either ex vivo or through a high-fat diet. Membrane cholesterol enrichment ex vivo led to a significant reduction in all P2X7-induced cellular responses in T cells. Importantly, diet-induced hypercholesterolemia in B6 mice was also associated with decreased sensitivity to ATP in CD4^+^ and CD8^+^ T cells, highlighting the relationship between cholesterol intake and the amplitudes of P2X7-induced cellular responses in T cells.

## 1. Introduction

The P2X purinergic receptor 7 (P2X7) is an ATP-gated cation channel that can trigger numerous cellular and physiologic functions [1]. The types and amplitudes of P2X7-induced cellular responses depend on the duration of stimulation by ≥150 µM of extracellular ATP (eATP) in its tetraanionic form [2]. Short-term stimulation of P2X7 induces the opening of ion channels [3] and phosphatidylserine (PS) externalization to the outer leaflet of the plasma membrane [4]. Long-term stimulation of P2X7 results in the formation of non-selective membrane pores, allowing entry of molecules with molecular masses of up to 900 Da [5,6]. Continuous stimulation with eATP can lead to cell death by apoptosis [7,8] or necrosis [9], depending on the cell type. In contrast, P2X7 activation may trigger an antiapoptotic or mitogenic activity [10,11,12].

P2X7 plays a major role in various immune processes, such as the maturation of proinflammatory cytokines [13], the proteolytic cleavage of transmembrane protein such as CD62L and TNFα [14,15], and the activation of T cells [15]. We previously showed that the regulation of the sensitivity of CD4^+^ and CD8^+^ T cells to eATP depends on their stage of activation and differentiation [16,17]. Moreover, we found that pathogenic B220^+^ CD4^−^CD8^−^ (DN) T cells that accumulate in lupus MRL/*lpr* and B6/*lpr* mice exhibit a large reduction in P2X7 membrane expression and sensitivity to eATP [18]. The reduced sensitivity of pathogenic B220^+^ DN T cells, but not normal B220^−^ T cells (either CD4^+^ or CD8^+^), to eATP could be due to the expression of a negative regulator of P2X7 that is still undefined. Importantly, approximately 75% of the MRL genome is derived from the LG/J mouse strain, which is characterized by large body size, rapid growth, elevated body weight, and high susceptibility to diet-induced obesity and atherosclerosis (https://www.jax.org/strain/000486, accessed on 4 May 2022). Therefore, we suggest that metabolic dysregulation could lead to reduced sensitivity of P2X7 to eATP. Indeed, lipid environment through its influence on the plasma membrane composition is known to affect P2X7 functionality [1]. Membrane cholesterol plays a critical role in regulating the activity of P2X7, which contains several putative cholesterol recognition amino acid consensus motifs within its N-terminal and proximal C-terminal regions [1]. The association of P2X7 with cholesterol-enriched microdomains/lipid rafts of the plasma membrane has been reported in different cell types [19]. The trafficking of P2X7 into lipid rafts seems to require caveolin-1 interaction or palmitoylation, depending on the cell type [20,21]. Palmitoylation of the cytoplasmic cysteine-rich region at the end of the second transmembrane domain is also involved in the lack of desensitization of P2X7 after eATP binding [22] and pore formation [23]. Moreover, phosphatidylglycerol and sphingomyelin were found to facilitate P2X7 pore formation [23]. In contrast, membrane cholesterol negatively regulates P2X7-mediated pore formation and cell death [24]. However, the impact of membrane cholesterol content on eATP-induced cellular functions had been extensively evaluated in P2X7-transfected fibroblast or HEK 293 cells and noticeably less in T cells.

The physiological and pathological importance of cholesterol metabolism in T-cell immunity has been widely demonstrated [25]. For example, the immunological synapse formed between a T cell and an antigen-presenting cell results from the aggregation of lipid rafts [26], plasma membrane microdomains enriched in cholesterol that dissipate when membrane cholesterol is depleted [27,28,29,30]. We recently showed that membrane cholesterol levels have a strong impact on cellular responses induced by the stimulation of eATP-gated P2X7 in T cells. Indeed, the modulation of membrane cholesterol level (either positively or negatively) profoundly altered the functionality of the P2X7 receptor expressed by the mouse leukemic EL4 and L1210 T-cell lines, which were either sensitive or resistant to eATP in the basal state. Thus, we were able to restore the ability of eATP to induce the opening of calcium ion channels and the formation of non-selective pores in L1210 T cells by reducing their membrane cholesterol level using methyl-β-cyclodextrin (MBCD). Conversely, we were able to inhibit the ability of EL4 cells to open calcium ion channels and form non-selective pores following stimulation with eATP by increasing the levels of membrane cholesterol using MBCD/cholesterol (MBCD/Chol) complexes [29]. In the present study, we extended these findings to CD4^+^ and CD8^+^ T cells from B6 mice. In accordance with our data obtained in leukemic T-cell lines, CD4^+^ and CD8^+^ normal T cells displayed a significantly reduced ability to form non-selective P2X7 pores and shed CD62L molecules when their levels of plasma membrane cholesterol were increased ex vivo. Moreover, we assessed the outcome of a high-fat/cholesterol (HFC) diet-induced hypercholesterolemia on the plasma membrane cholesterol content and the functionality of P2X7 in CD4^+^ and CD8^+^ T cells. Importantly, in B6 mice fed the HFC diet, the high levels of circulating cholesterol were associated with decreased sensitivity to eATP in CD4^+^ and CD8^+^ T cells, highlighting for the first time a relationship between cholesterol intake and P2X7 functionality in T cells. We also show herein that pore formation and CD62L shedding are not similarly affected by plasma membrane cholesterol. Indeed, pore formation followed a dose-dependent effect, whereas CD62L shedding was highly sensitive to cholesterol modulation, indicating that P2X7-mediated cellular responses are not regulated in an all-or-none manner by plasma membrane cholesterol content. 

## 2. Results

### 2.1. Plasma Membrane Levels of Cholesterol and GM1 Gangliosides in T Cells at Different Stages of Differentiation and the Consequences on Their Sensitivity to eATP

**Figure 1:**
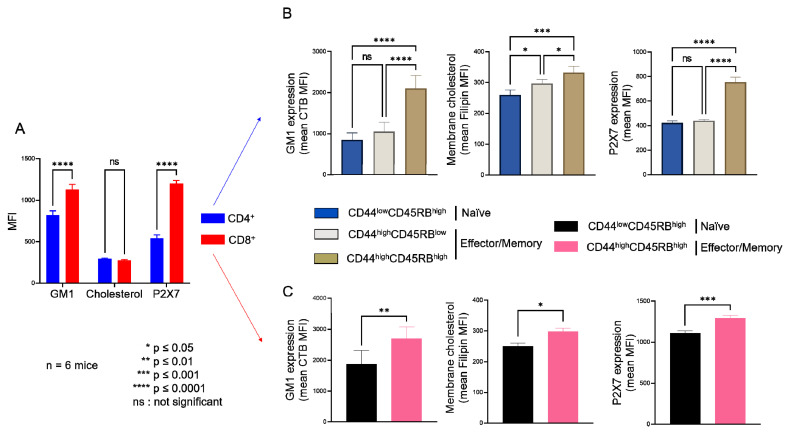
**Plasma membrane levels of GM1 gangliosides, cholesterol and P2X7 receptor in T cells at different stages of differentiation.** Plasma membrane levels of GM1, cholesterol, and P2X7 were quantified by flow cytometry using FITC-conjugated cholera toxin B subunit (CTB), filipin III, and anti-P2X7 extracellular domain mAb, respectively. Results are presented as the mean MFI values for GM1, cholesterol, and P2X7 in the CD4^+^ and CD8^+^ T-cell subpopulations (**A**) and in CD44^low^CD45RB^high^ naïve and CD44^high^CD45RB^low^ or CD44^high^CD45RB^high^ effector/memory CD4^+^ (**B**) and CD8^+^ (**C**) T-cell subsets. Data are expressed as the mean of percentages (±SEM, n ≥ 6 mice). Asterisks denote statistically significant differences * *p* ≤ 0.05; ** *p* ≤ 0.01; *** *p* ≤ 0.001 **** *p* ≤ 0.0001; ns: not significant.

**Figure 2:**
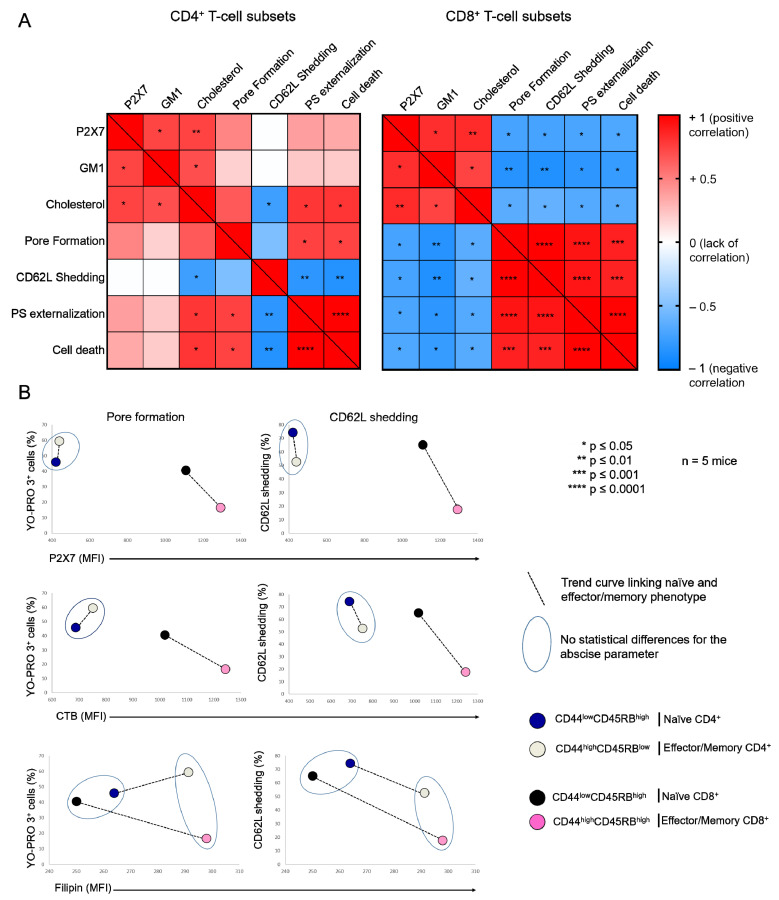
**Relationship between the plasma membrane levels of GM1, cholesterol, and P2X7, and cellular responses induced by eATP in T-cell subsets**. (**A**) Pearson correlation matrix showing the degree of correlation between the plasma membrane levels of GM1, cholesterol, and P2X7, pore formation, CD62L shedding, PS externalization and cell death in CD4^+^ and CD8^+^ T-cell subpopulations in the presence of 500 µM ATP for 30 min at 37 °C. The magnitude and direction of the correlation coefficient is indicated on a color gradient that varies from +1 (positive correlation) to −1 (negative correlation). White squares indicate the absence of a correlation. Red and blue squares represent positive and negative correlations between two variables, respectively. Statistically significant correlations are denoted by asterisks within squares: * *p* ≤ 0.05, ** *p* ≤ 0.01, *** *p* ≤ 0.001, **** *p* ≤ 0.0001. (**B**) Scatter plots showing the phenotypic profile of CD44^low^CD45RB^high^ naïve and CD44^high^CD45RB^low^ or CD44^high^CD45RB^high^ effector/memory T-cell subsets (either CD4^+^ or CD8^+^) in terms of the amplitude of ATP-induced pore formation and CD62L shedding (500 µM, 30 min, 37 °C) and the plasma membrane levels of GM1, cholesterol, and P2X7. The T-cell subsets that were non-statistically different for the abscissa parameter (either GM1, cholesterol, or P2X7 expression) are surrounded by a blue ellipse. The black dotted line links the naïve subset (either CD4^+^ or CD8^+^) with its effector/memory counterpart. Data are expressed as the mean of percentages (n ≥ 5 mice).

**Figure A1:**
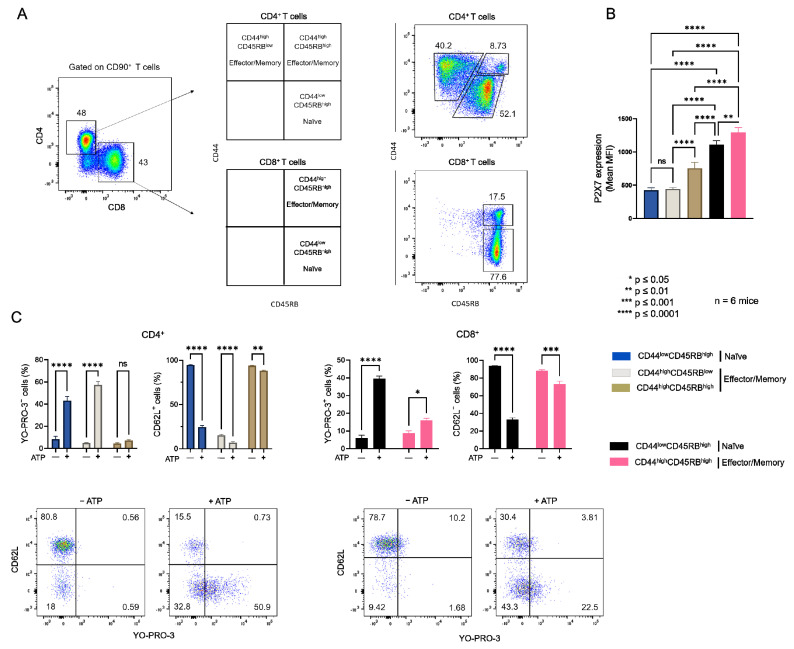
**P2X7-mediated cellular responses depend on the differentiation stage of CD4^+^ and CD8^+^ T cells.** (**A**) Gating strategy for the identification of CD44^low^CD45RB^high^ naive and CD44^high^CD45RB^high^ or CD44^low^CD45RB^high^ effector/memory CD4^+^ and CD8^+^ T-cell subsets from mouse spleens. (**B**) Plasma membrane levels of P2X7 was quantified by flow cytometry using BV605-conjugated anti-P2X7 extracellular domain mAb. (**C**) P2X7-dependent pore formation (left panel) and shedding of CD62L (right panel) were evaluated for the various stages of differentiation of CD4^+^ and CD8^+^ T cells following stimulation with 500 µM eATP for 30 min at 37 °C. Results are presented as mean histograms and dot plots representative of the experiments. Data are expressed as the mean of percentages (±SEM, n ≥ 6 mice). Asterisks denote statistically significant differences * *p* ≤ 0.05, ** *p* ≤ 0.01, *** *p* ≤ 0.001, **** *p* ≤ 0.0001, ns: not significant.

As we previously reported [16,17], the sensitivity of CD4^+^ and CD8^+^ T cells from B6 mice to eATP depends more on their stage of differentiation than the levels of P2X7 plasma membrane expression (Figure A1). Indeed, we showed that CD44^low^CD45RB^high^ naïve T cells (either CD4^+^ or CD8^+^) efficiently shed their homing CD62L molecules and form non-selective P2X7 pores, whereas CD44^high^CD45RB^low^ and CD44^high^CD45RB^high^ effector/memory T cells (either CD4^+^ or CD8^+^) were unable to shed CD62L and, to a lesser extent, form non-selective P2X7 pores, although they express higher levels of P2X7 (Figure A1). Similarly, we recently showed that the EL4 and L1210 leukemic T cell lines display similar high levels of P2X7 membrane expression but an opposite ability to trigger channel opening, pore formation, and phosphatidyserine exposure upon eATP stimulation. Importantly, we have observed that cholesterol and GM1 gangliosides levels at the plasma membrane play a central role in the sensitivity of EL4 and L1210 T cells to eATP [29]. The well-known role of cholesterol and GM1 gangliosides in the formation, maintenance, and aggregation of lipid rafts [31,32] prompted us to evaluate the levels of plasma membrane cholesterol and GM1 in naïve and effector/memory CD4^+^ and CD8^+^ T-cell subsets from B6 mice (Figure 1). In addition, we performed correlation analyses to gain insights into the respective roles of GM1 and cholesterol plasma membrane levels, as well as that of P2X7 membrane expression, in the sensitivity of naïve and effector/memory T-cell subsets (either CD4^+^ or CD8^+^) to eATP stimulation (Figure 2).

Thus, flow cytometry analyses of filipin staining showed CD4^+^ and CD8^+^ T cells to display similar levels of plasma membrane cholesterol, whereas significantly higher levels of GM1 (FITC-CTB labelling) and P2X7 were expressed by CD8^+^ than CD4^+^ T cells (Figure 1A). Among CD4^+^ T-cell subsets, the CD44^low^CD45RB^high^ naïve and CD44^high^CD45RB^low^ effector/memory subsets showed lower levels of plasma membrane GM1, cholesterol, and P2X7 than the very minor (3.45% ± 0.57) CD44^high^CD45RB^high^ effector/memory subset (Figure 1B). Similarly, among CD8^+^ T cells, the CD44^low^CD45RB^high^ naïve subset showed lower levels of plasma membrane GM1, cholesterol, and P2X7 than the CD44^high^CD45RB^high^ effector/memory subset (Figure 1C). As the CD4^+^ and CD8^+^ T-cell subsets displayed significantly different sensitivity to P2X7-induced cellular responses (Figure A1), we investigated a possible relationship between phenotypic (cholesterol, GM1, and P2X7 membrane levels) and functional P2X7 characteristics (pore formation, CD62L shedding, PS externalization, cell death) in CD4^+^ and CD8^+^ T-cell subpopulations by creating a matrix showing the correlation coefficient between these variables (Figure 2). For CD8^+^ T cells, the phenotypic characteristics positively correlated with each other but negatively correlated with functional P2X7 characteristics. Moreover, all P2X7-induced cellular responses positively correlated with each other, suggesting that they are regulated in a similar manner in CD8^+^ T cells (Figure 2A). Interestingly, for the CD4^+^ T-cell subpopulation, P2X7-induced cellular responses correlated with plasma membrane cholesterol content only. Moreover, cholesterol content negatively affected CD62L shedding but positively affected pore formation, PS externalization, and cell death (Figure 2A). Therefore, our data suggest that P2X7-induced cellular function are regulated differently in CD4^+^ and CD8^+^ T cells. We generated scatter plots comparing the levels of pore formation or CD62L shedding according to P2X7, GM1, or cholesterol plasma membrane content (Figure 2B) to obtain insights into the relationship between the functional and phenotypic characteristics expressed by the various CD4^+^ and CD8^+^ T-cells subsets (either naïve or effector/memory). In accordance with the correlation matrix analyses, the sensitivity of the CD8^+^ T-cell subsets to eATP negatively correlated with all phenotypic parameters. Indeed, CD44^high^CD45RB^high^ effector/memory CD8^+^ T cells, which showed higher levels of plasma membrane P2X7, GM1, and cholesterol than their CD44^low^CD45RB^high^ naïve counterpart, displayed the lowest capacity to form pore and shed CD62L. By contrast, CD44^low^CD45RB^high^ naïve and CD44^high^CD45RB^low^ effector/memory CD4^+^ T cells, although displaying similar levels of P2X7 and GM1, expressed P2X7-induced cellular responses of significantly different amplitude. Indeed, CD62L shedding was significantly more efficient than pore formation in naïve CD4^+^ T cells than in their effector/memory counterpart. Our data suggest a limited influence of P2X7 and GM1 levels on P2X7-induced cellular responses in CD4^+^ T cells. Surprisingly, the levels of plasma membrane cholesterol were similarly low in naïve CD4^+^ and CD8^+^ T cells and high in their effector/memory counterpart (Figure 2B). Moreover, although an increase in cholesterol content was associated with a decrease in both pore formation and CD62L shedding in CD8^+^ T cells, it was associated with an increase in pore formation and a decrease in CD62L shedding in CD4^+^ T cells. Altogether, our data suggest that cholesterol content plays a key role in the amplitude of P2X7-induced cellular responses in T-cells.

### 2.2. Amplitude of P2X7-Induced Cellular Responses in T Cells Following Plasma Membrane Cholesterol Modulation

**Figure 3:**
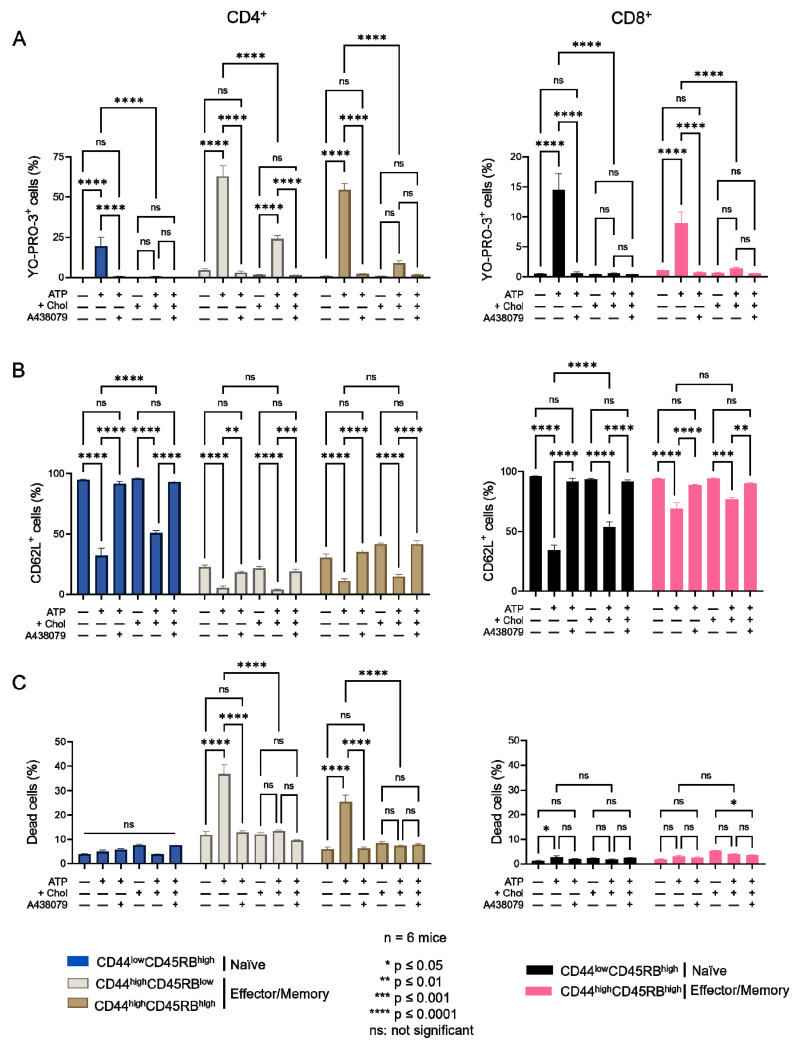
**Amplitude of P2X7-induced cellular responses in T cells following plasma membrane cholesterol modulation.** Amplitude of P2X7-mediated pore formation (**A**), CD62L shedding (**B**) and cell death (**C**) were evaluated among CD44^low^CD45RB^high^ naïve and CD44^high^CD45RB^low^ or CD44^high^CD45RB^high^ effector/memory CD4^+^ and CD8^+^ T-cell subsets in the presence of 500 µM eATP for 30 min at 37 °C with or without pre-treatment with 10 µM of the P2X7 antagonist A438079 (+A438079), following ex vivo plasma membrane cholesterol enrichment using MBCD/Chol complexes. Data are expressed as the mean of percentages (±SEM, n ≥ 6 mice). Asterisks denote statistically significant differences * *p* ≤ 0.05, ** *p* ≤ 0.01, *** *p* ≤ 0.001, **** *p* ≤ 0.0001, ns: not significant.

**Figure A2:**
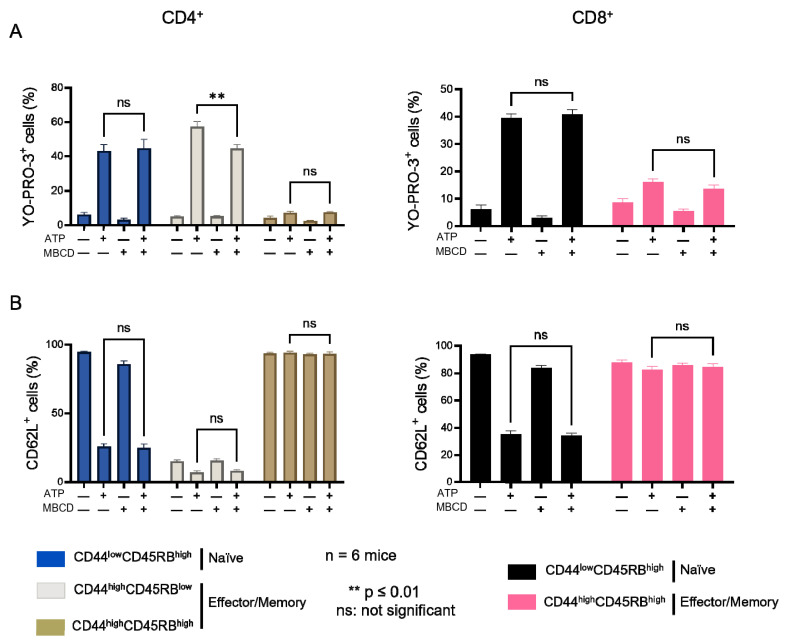
Amplitude of P2X7-induced cellular responses in T cells following plasma membrane cholesterol depletion by MBCD. Amplitude of P2X7-mediated pore formation (**A**) and CD62L shedding (**B**) were evaluated among CD44^low^CD45RB^high^ naïve and CD44^high^CD45RB^low^ or CD44^high^CD45RB^high^ effector/memory CD4^+^ and CD8^+^ T-cell subsets in the presence of 500 µM eATP for 30 min at 37 °C following ex vivo plasma membrane cholesterol depletion using 5 mM MBCD for 20 min at 37 °C. Data are expressed as the mean of percentages (±SEM, n ≥ 6 mice). Asterisks denote statistically significant differences ** *p* ≤ 0.01, ns: not significant.

We next wished to confirm the involvement of cholesterol in the amplitude of P2X7-induced cellular responses in CD4^+^ and CD8^+^ T cells. We thus modulated their plasma membrane cholesterol content ex vivo by MBCD and MBCD/Chol treatment and in vivo by HFC diet. When we treated spleen cells with 5 mM MBCD for 20 min at 37 °C, more than 90% of both naïve and effector/memory CD4^+^ and CD8^+^ T cells were still viable after treatment. Unexpectedly, cholesterol depletion from the plasma membrane of splenocytes had no significant effect on the sensitivity of T cells to eATP, regardless of the stage of differentiation (naïve, effector/memory) of the CD4^+^ and CD8^+^ T cells (Figure A2). By contrast, increasing plasma membrane cholesterol levels in splenocytes from B6 mice using MBCD/Chol complexes resulted in a strong reduction in the capacity of both naïve and effector/memory CD4^+^ and CD8^+^ T cells to form the P2X7-mediated pores in response to stimulation with 500 μM eATP (Figure 3). Moreover, naïve T cells (either CD4^+^ or CD8^+^) showed a greater level of reduction in P2X7-mediated pore formation than their CD44^high^CD45RB^high^ effector/memory counterparts following MBCD/Chol treatment (Figure 3A). Importantly, MBCD/Chol treatment also altered the ability of naïve CD4^+^ and CD8^+^ T cells to shed CD62L molecules in response to 500µM eATP (Figure 3B). Finally, effector/memory CD4^+^ T cells, known to be much more responsive to eATP-induced cell death than CD8^+^ and naïve CD4^+^ T cells, displayed a significantly reduced sensitivity to cell death following plasma membrane cholesterol enrichment (Figure 3C). ATP-induced cellular responses could be prevented by the P2X7 inhibitor A438079 (Figure 3), confirming the specificity of pore formation, CD62L shedding, and cell death. Altogether, our findings confirm that plasma membrane cholesterol levels are a key factor in modulating the amplitude of P2X7-induced cellular function in CD4^+^ and CD8^+^ T cells (either naïve or effector/memory).

### 2.3. Diet-Induced Hypercholesterolemia Increase Effector/Memory CD4^+^ and CD8^+^ T Cell Numbers in B6 Mice

**Figure 4:**
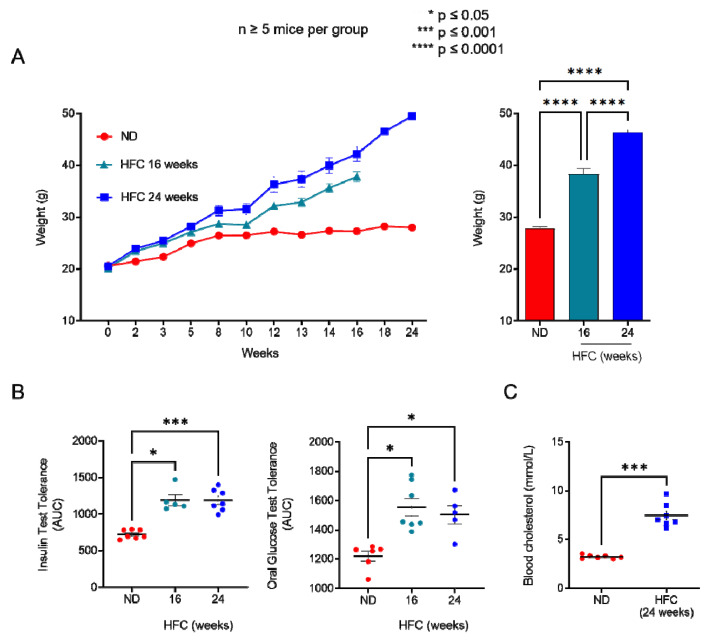
**Diet-induced hypercholesterolemia in B6 mice.** (**A**) Mean body weight of mice fed either a normal diet (ND) or a high-fat diet (HFC) presented as the weekly evolution (left panel) or following 16 or 24 weeks of the diet (right panel). (**B**) Insulin tolerance test (ITT, left panel) and oral glucose tolerance test (OGTT, right panel) performed on mice fed either a normal diet or a high-fat diet maintained for 16 and 24 weeks. (**C**) Level of circulating cholesterol in the blood of mice fed either a normal diet or a high-fat diet maintained for 24 weeks. Data are expressed as the mean of percentages (±SEM, n ≥ 5 mice). Asterisks denote statistically significant differences: * *p* ≤ 0.05, *** *p* ≤ 0.001, **** *p* ≤ 0.0001.

**Figure 5:**
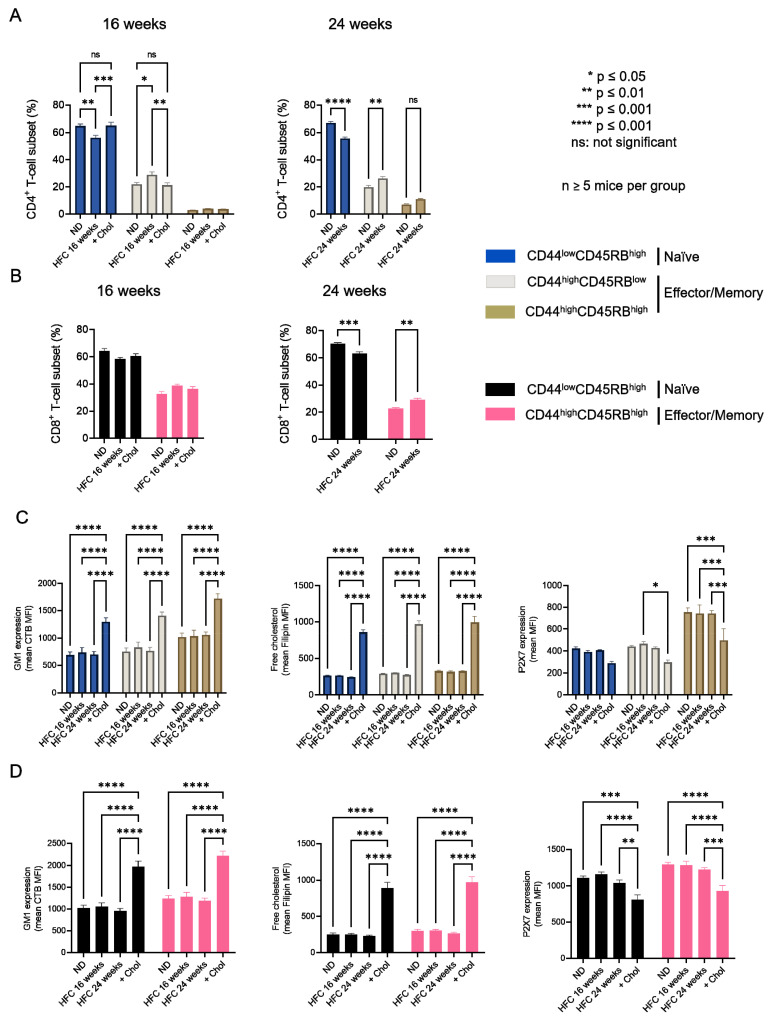
**Impact of diet-induced hypercholesterolemia on the proportion of CD4^+^ and CD8^+^ T-cell subsets in B6 mice**. Percentage of CD4^+^ (**A**) and CD8^+^ (**B**) T-cell subsets quantified by flow cytometry for mice fed either a normal diet (ND) or a high-fat diet (HFC) maintained for 16 (left panel) or 24 weeks (right panel). Splenocytes from mice fed a normal diet were treated with MBCD/Chol complexes (+Chol) ex vivo as a positive control for cholesterol enrichment. (**C,D**) Plasma membrane levels of GM1, cholesterol, and P2X7 quantified by flow cytometry in splenocytes from mice fed either a normal diet or a high-fat diet maintained for 16 (left panel) or 24 weeks (right panel). Results are presented as the mean MFI values for GM1, cholesterol, and P2X7 among CD44^low^CD45RB^high^ naïve and CD44^high^CD45RB^low^ or CD44^high^CD45RB^high^ effector/memory CD4^+^ (**C**) and CD8^+^ (**D**) T-cell subsets. Data are expressed as the mean of percentages (±SEM, n ≥ 5 mice). Asterisks denote statistically significant differences: * *p* ≤ 0.05, ** *p* ≤ 0.01, *** *p* ≤ 0.001, **** *p* ≤ 0.0001, ns: not significant.

We showed that increasing membrane cholesterol levels ex vivo negatively affects T-cell sensitivity to eATP (Figure 3). We therefore evaluated whether diet-induced hypercholesterolemia in B6 mice can modulate the amplitude of P2X7-mediated pore formation and CD62L shedding in CD4^+^ and CD8^+^ T cells at different stages of differentiation. Two independent experiments were performed for either 16 or 24 weeks, in which male B6 mice were fed a normal diet, or a diet enriched in lipids and supplemented with cholesterol (HFC diet) (Figure 4). In both experiments, HFC-fed mice displayed a marked increase in body weight to reach 38 ± 0.13 g after 16 and 46 ± 0.18 g after 24 weeks of the diet, versus 27 ± 0.1 g for mice receiving a normal diet (Figure 4A), along with a significant alteration in their ability to regulate glycaemia due to insulin resistance (Figure 4B). Importantly, HFC-fed mice showed a significant increase in circulating cholesterol levels, leading to chronic hypercholesterolemia, as shown by cholesterol blood measurements following a 24 h fast (Figure 4C). Interestingly, such metabolic dysregulation was associated with a decrease in the size of the CD44^low^CD45RB^high^ naïve CD4^+^ T-cell subset and an increase in that of their CD44^high^CD45RB^low^ effector/memory counterpart (Figure 5A). This increase was already significant after 16 weeks of the diet and became even more marked when the diet was extended to 24 weeks (Figure 5A). Unexpectedly, 24 weeks of the diet were required to significantly increase the numbers of CD44^high^CD45RB^high^ effector/memory T cells in the CD8^+^ T-cell subpopulation (Figure 5B). We ascertained that the modification in the ratio between naïve and effector/memory T-cells resulted from metabolic changes induced by the HFC diet and not by direct alteration of the levels of CD44 and CD45RB membrane expression due to plasma membrane remodeling. Indeed, the mean fluorescence intensity (MFI) of CD44 and CD45RB membrane expression quantified by flow cytometry was not affected by cholesterol enrichment in splenocytes from mice fed a normal diet treated with MBCD/Chol complexes ex vivo (data not shown). Moreover, MBCD/Chol treatment did not influence the ratio between the naïve and effector/memory phenotypes of either CD4^+^ or CD8^+^ T cell subpopulations relative to untreated splenocytes (Figure 5A,B), confirming the effect observed following the HFC diet. We further evaluated the effect of the HFC diet on membrane remodeling by evaluating the membrane levels of the lipid raft marker GM1, cholesterol, and P2X7 receptor. Surprisingly, neither 16 or 24 weeks of the HFC diet significantly modified the membrane levels of GM1, cholesterol, or P2X7 in T cells (either CD4^+^ or CD8^+^) (Figure 5C). However, ex vivo treatment with MBCD/Chol complexes, well known to induce a massive increase in plasma membrane cholesterol content, resulted in a significant increase in both GM1 and cholesterol levels in the plasma membranes of CD4^+^ and CD8^+^ T cells regardless of their state of differentiation (Figure 5C). It is worth noting that treatment with MBCD/Chol complexes resulted in a significant decrease in P2X7 membrane expression by both naïve and effector/memory CD8^+^ T cells as well as by CD44^high^CD45RB^high^ effector/memory CD4^+^ T cells (Figure 5C, right panel). Altogether, our data suggest that diet-induced hypercholesterolemia was not sufficient to significantly modify the levels of GM1, cholesterol, or P2X7 at the T-cell plasma membrane. However, we cannot exclude the possibility that there were slight modifications that were not detectable using our fluorescent probes.

### 2.4. Diet-Induced Hypercholesterolemia Affected P2X7-Induced Cellular Responses in Naive and Effector/Memory CD4^+^ and CD8^+^ T Cells

**Figure 6:**
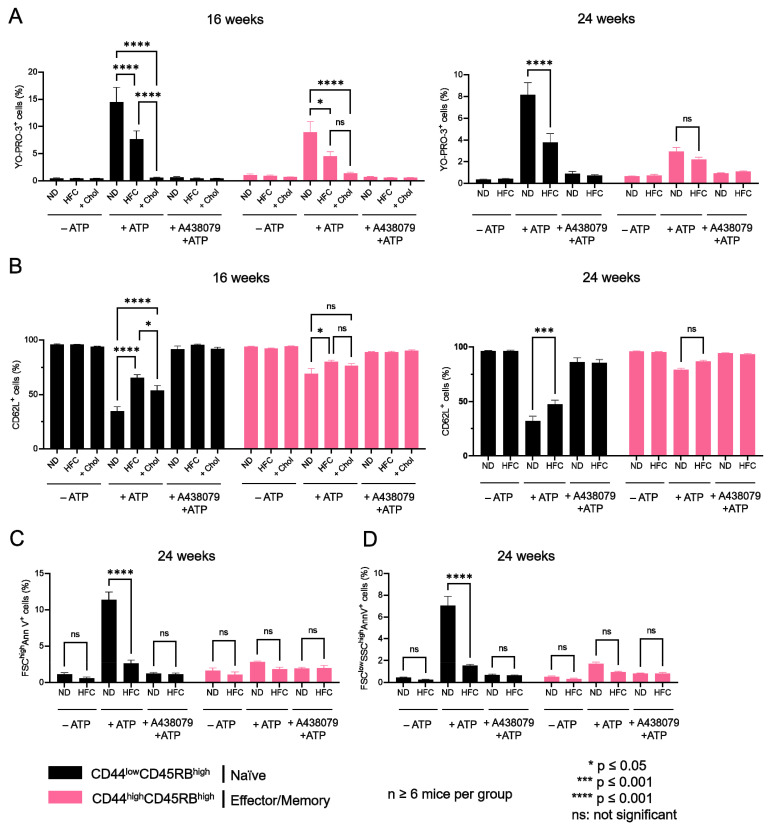
**Diet-induced hypercholesterolemia affects P2X7-induced cellular responses in naive and effector/memory CD8^+^ T cells.** Following stimulation with 500 µM eATP for 30 min at 37 °C with or without pre-treatment with 10 µM of the P2X7 antagonist A438079 (+A438079), P2X7-dependent pore formation (**A**), CD62L shedding (**B**), reversible PS externalization in living cells (FSC^high^AnnV^+^) (**C**), and cell death characterized by irreversible PS externalisation (FSC^low^SSC^high^AnnV^+^) (**D**) were evaluated in CD44^low^CD45RB^high^ naive and CD44^high^CD45RB^high^ effector/memory CD8^+^ T cells from mice fed either a normal diet (ND) or a high-fat diet (HFC) maintained for 16 (left panel) or 24 weeks (right panel). Splenocytes from mice fed a normal diet were treated with MBCD/Chol complexes (+Chol) ex vivo as a positive control for cholesterol enrichment. Data are expressed as the mean of percentages (±SEM, n ≥ 6 mice). Asterisks denote statistically significant differences * *p* ≤ 0.05, *** *p* ≤ 0.001, **** *p* ≤ 0.0001, ns: not significant.

**Figure 7:**
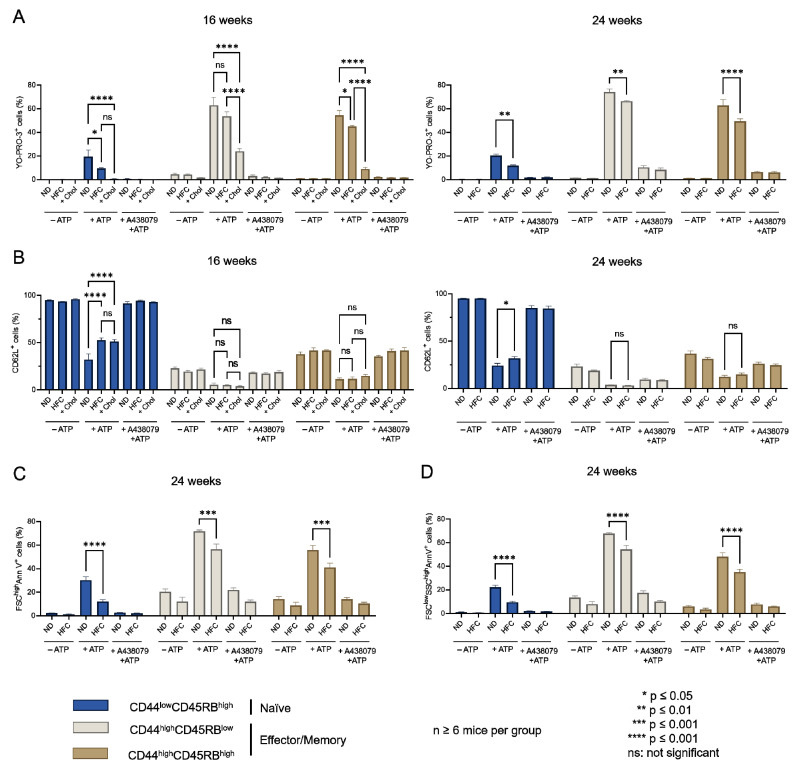
**Diet-induced hypercholesterolemia affects P2X7-induced cellular responses in naive and effector/memory CD4^+^ T cells.** Following stimulation with 500 µM eATP for 30 min at 37 °C with or without pre-treatment with 10 µM of the P2X7 antagonist A438079 (+A438079), P2X7-dependent pore formation (**A**), CD62L shedding (**B**), reversible PS externalization in living cells (FSC^high^AnnV^+^) (**C**), and cell death characterized by irreversible PS externalisation (FSC^low^SSC^high^AnnV^+^) (**D**) were evaluated in CD44^low^CD45RB^high^ naive and CD44^high^CD45RB^high^ and CD44^high^CD45RB^low^ effector/memory CD4^+^ T cells from mice fed either a normal diet (ND) or a high-fat diet (HFC) maintained for 16 (left panel) or 24 weeks (right panel). Splenocytes from normal diet-fed mice were treated with MBCD/Chol complexes (+Chol) ex vivo as positive control of cholesterol enrichment. Data are expressed as the mean of percentages (±SEM, n ≥ 6 mice). Asterisks denote statistically significant differences * *p* ≤ 0.05; ** *p* ≤ 0.01; *** *p* ≤ 0.001, **** *p* ≤ 0.0001, ns: not significant.

**Figure A3:**
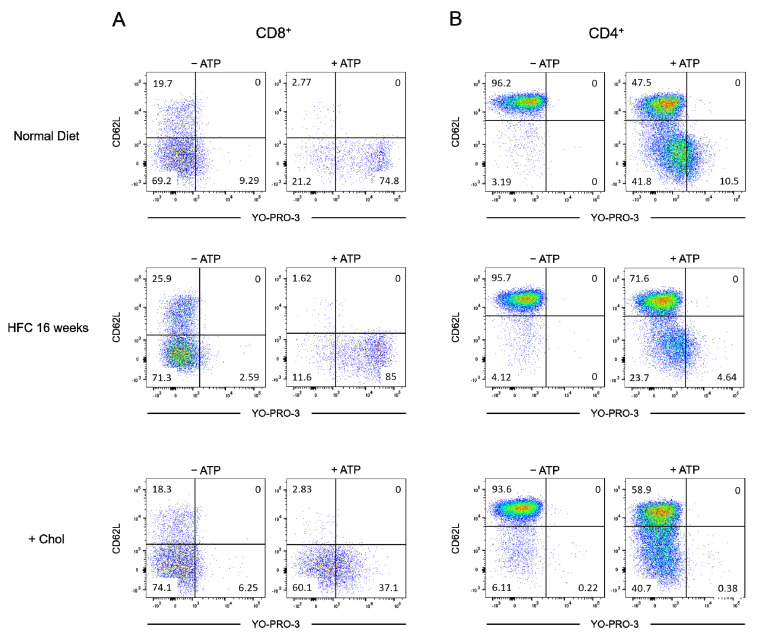
**Diet-induced hypercholesterolemia affects P2X7-induced cellular responses in naive and effector/memory CD8^+^ and CD4^+^ T cells**. Flow cytometry dot plots representative of the P2X7-induced cellular function assay performed on CD8^+^ (**A**) and CD4^+^ (**B**) T-cell subsets from mice fed either a normal diet (ND) or a high-fat diet (HFC) maintained for 16 weeks or on splenocytes from normal fed mice treated ex vivo with MBCD/Chol complexes (+Chol).

The influence of diet-induced hypercholesterolemia on P2X7-induced cellular responses was evaluated in CD4^+^ and CD8^+^ T-cell subsets from mice fed either a normal diet or a HFC diet for 16 and 24 weeks at various differentiation stages by their ability to form non-selective P2X7 pores, cleave homing CD62L molecules, externalize PS, and enter cell death following stimulation with 500 µM eATP (Figure 6, Figure 7 and Figure A3). We treated splenocytes from mice receiving a normal diet with MBCD/Chol complexes ex vivo as a positive control of the cholesterol-dependent inhibition of P2X7-induced cellular responses in T cells. Such massive cholesterol enrichment led to the strong inhibition of all P2X7-induced cellular responses in CD4^+^ and CD8^+^ T cells (either naïve or effector/memory) (Figure 6 and Figure 7). Interestingly, the naïve CD8^+^ T-cell subset showed a significantly reduced ability to form P2X7-mediated pore following 16 weeks of the HFC diet, whereas 24 weeks of HFC diet were required to observe a similar inhibition of pore formation in their naïve and effector/memory CD4^+^ T-cell counterpart (Figure 6A and Figure 7A). Moreover, pore formation was modulated in a dose-dependent manner as inhibition was higher following 24 rather than 16 weeks of the HFC diet and even stronger after ex vivo MBCD/Chol complexes treatment. Among P2X7-induced cellular responses, CD62L shedding displayed the highest sensitivity to plasma membrane cholesterol content. Indeed, 16 weeks of the HFC diet was sufficient to significantly inhibit the ability of naïve CD8^+^ and CD4^+^ T cells to shed CD62L molecules (Figure 6B and Figure 7B). In addition, 24 weeks of the HFC diet or MBCD/Chol treatment ex vivo did not amplify the inhibition of P2X7-induced CD62L shedding, suggesting that maximum inhibition was already reached after 16 weeks (Figure 6B and Figure 7B). Moreover, the stimulation of CD4^+^ and CD8^+^ T cell subsets from B6 mice fed a normal diet with 500 µM eATP for 30 min induced the externalization of PS, either in a reversible manner or associated with a decrease in cell size (FSC^low^) and increase in cell granularity (SSC^high^), characteristics of dying cells. Interestingly, after 24 weeks of the HFC diet, living FSC^high^SSC^low^ CD8^+^ and CD4^+^ T cells displayed a significant reduction in their capacity to externalize PS, regardless of their stage of differentiation, naïve or effector/memory (Figure 6C and Figure 7C). In accordance with our previous reports [16,17], under the condition of the normal diet, CD4^+^ T cells showed higher sensitivity to P2X7-induced cell death than CD8^+^ T cells (Figure 6D and Figure 7D). After 16 weeks of the HFC diet, CD4^+^ T cells displayed a similar sensitivity to cell death as these from mice fed a normal diet (data not shown). However, after 24 weeks of the HFC diet, the percentages of CD44^low^CD45RB^high^ naïve and both CD44^high^CD45RB^low^ and CD44^high^CD45RB^high^ effector/memory CD4^+^ T cells entering cell death significantly decreased following eATP stimulation (Figure 7D). Similarly, 24 weeks of the HFC diet significantly reduced the percentage of CD44^low^CD45RB^high^ naïve CD8^+^ T cells entering cell death following eATP stimulation (Figure 6D). It is worth noting that the CD44^high^CD45RB^high^ effector/memory CD8^+^ T cell subset, which display both the highest level of plasma membrane cholesterol and the lowest sensitivity to eATP among the T-cell subpopulations (Figure 1, Figure 2 and Figure 6), did not show any significant inhibition of P2X7-induced pore formation as well as CD62L shedding, PS externalization, or cell death following 16 or 24 weeks of the HFC diet (Figure 6). ATP induced cellular responses could be prevented by the P2X7 inhibitor A438079 (Figure 6 and Figure 7) confirming the specificity of pore formation, CD62L shedding, PS externalization, and cell death. Altogether, these studies confirm the relationship between cholesterol intake and P2X7-induced cellular responses. Moreover, we highlight that CD62L shedding is more sensitive to cholesterol modulation than pore formation. Finally, we show that increased circulating cholesterol is responsible for the reduction in T-cell sensitivity to eATP stimulation.

## 3. Discussion

Previously, we reported that the sensitivity of the CD4^+^ and CD8^+^ T-cell subpopulations to eATP depends more on their stage of activation and differentiation than on their levels of membrane P2X7 [16,17]. Recently, we also showed that the sensitivity of leukemic T-cell lines to eATP is directly affected by their membrane cholesterol content and, therefore, by the localization of P2X7 within cholesterol-enriched microdomains [29]. Moreover, various studies have reported that plasma membrane cholesterol levels significantly fluctuate during the activation and differentiation of normal T cells [33]. Thus, T-cell activation through TCR signaling is highly dependent on plasma membrane cholesterol levels, well known to control immunological synapse formation through lipid raft aggregation [26]. In the present study, which is focused on the understanding of P2X7 signaling at the T-cell membrane, we therefore investigated whether there is a link between plasma membrane cholesterol levels and the cellular responses mediated by P2X7 in naïve and effector/memory CD4^+^ and CD8^+^ T-cell subsets from B6 mice. BzATP is often used as a ligand of P2X7. However, it has been reported that BzATP triggers pore formation in lymphocytes with an EC50 value of about 15 µM compared to 85 µM for ATP. Thus, BzATP stimulates P2X7-induced pore formation up to 30% more than ATP [34]. Therefore, in all our experiments, splenocytes have been activated with the physiological agonist ATP, instead of BzATP, to avoid an overstimulation of P2X7R-mediated cellular responses, which could have masked potential differences between T-cell subsets (either CD4^+^ or CD8^+^). For instance, the effector/memory CD44highCD45RBlow CD4+ T-cell subset, which is particularly sensitive to ATP-induced cell death [16,17], is rapidly lost following BzATP stimulation, rendering challenging the comparison with other T-cell subset. For the first time, we highlight a negative correlation between P2X7-triggered cellular responses (i.e., pore formation, CD62L shedding, PS externalization, cell death) and the levels of plasma membrane cholesterol and the raft markers GM1 gangliosides in normal T cells. Indeed, CD44^low^CD45RB^high^ naïve and CD44^high^CD45RB^low^ effector/memory CD4^+^ T-cell subsets from B6 mice showed high sensitivity to eATP, along with low levels of plasma membrane cholesterol and GM1 gangliosides. By contrast, CD44^high^CD45RB^high^ effector/memory CD4^+^ subsets, as well as CD44^low^CD45RB^high^ naïve and CD44^high^CD45RB^high^ effector/memory CD8^+^ T-cell subsets, showed poor sensitivity to eATP stimulation and high levels of both plasma membrane cholesterol and GM1 gangliosides. Importantly, and in agreement with our previous reports [16,17], these CD4^+^ and CD8^+^ T-cell subsets showed higher levels of membrane P2X7 than highly eATP responsive CD44^low^CD45RB^high^ naïve and CD44^high^CD45RB^low^ effector/memory CD4^+^ T-cell subsets. Overall, our data suggest that plasma membrane cholesterol and lipid rafts play a key role in the amplitude of P2X7-induced cellular responses in T cells. Therefore, we next investigated whether the modulation of plasma membrane cholesterol content can significantly affect the functionality of P2X7 in naïve and effector/memory CD4^+^ and CD8^+^ T-cell subsets from B6 mice. Two experimental approaches were followed in the present study to modulate plasma membrane cholesterol content in T cells: (1) in vitro cholesterol depletion or loading using MBCD either alone or complexed with cholesterol; (2) in vivo diet-induced hypercholesterolemia. The use of MBCD to efficiently modulate plasma membrane cholesterol content in vitro has been validated by many studies [19,27,35]. Cholesterol depletion using MBCD alone did not allow us to significantly modify eATP sensitivity. This absence of an effect of cholesterol depletion could be explained by the presence of a large number of erythrocytes in the spleen-cell suspensions treated with MBCD, of which the plasma membrane is known to contain high levels of cholesterol. Thus, the MBCD molecules could have been saturated by erythrocyte cholesterol, rendering MBCD unable to reduce the cholesterol content in T cell plasma membrane. We did not eliminate erythrocytes from the spleen-cell suspension in our experiments because their lysis is well known to release large amounts of eATP, which non-specifically activates P2X7. On the contrary, plasma membrane cholesterol enrichment using MBCD/Chol complexes allowed a significant reduction in the amplitude of all P2X7-induced cellular responses in naïve and effector/memory CD4^+^ and CD8^+^ T-cell subsets, confirming the key role of plasma membrane cholesterol in the sensitivity of T cells to the eATP/P2X7 pathway. However, as the use of MBCD/Chol complexes is far from being physiological, we performed in vivo experiments using a high-fat diet. Thus, following 16 and 24 weeks of a HFC diet, B6 mice presented all the expected parameters of diet-induced obesity. In particular, they showed a massive increase in body weight (around 40%) and plasma levels of cholesterol (2-fold), as well as severe liver steatosis shown by histology analysis. They also showed impaired insulin sensitivity associated with glucose resistance. Therefore, our model was suitable for evaluating the link between P2X7 sensitivity in CD4^+^ and CD8^+^ T-cell subsets and increasing cholesterol levels in the plasma membrane due to diet-induced hypercholesterolemia. In parallel, we treated splenocytes from mice fed a normal diet using MBCD/Chol complexes ex vivo to ascertain that the reduction in P2X7 sensitivity of the CD4^+^ and CD8^+^ T cell subsets following the HFC diet was mainly dependent on plasma membrane cholesterol modulation and not the consequences of the modulation of various biological parameters linked to obesity. In addition, the MFI for CD44 and CD45RB differentiation markers was not affected in splenocytes treated with MBCD/Chol ex vivo, confirming that changes in the ratio between CD44^low^CD45RB^high^ naïve and CD4^+^CD44^high^CD45RB^low^ and CD8^+^CD44^high^CD45RB^high^ effector/memory T cells observed following the HFC diet resulted from metabolic changes and not from direct alterations of the levels of membrane CD44 and CD45RB due to plasma membrane remodeling. Indeed, diet-induced obesity in B6 mice has been extensively reported to be associated with a strong inflammatory response [36]. Moreover, MBCD/Chol treatment is also used as a control of maximum cholesterol loading of T cells due to its ability to increase plasma membrane cholesterol to a content that cannot be obtained through a HFC diet. Satisfyingly, the reduction in P2X7 sensitivity observed in CD4^+^ and CD8^+^ T cells from mice fed the HFC diet for 16 or 24 weeks showed a similar profile as that observed after ex vivo plasma membrane cholesterol enrichment using MBCD/Chol complexes, confirming that the effect of diet-induced obesity on T-cell sensitivity to eATP was mainly due to plasma membrane cholesterol enrichment. Moreover, these results highlight the positive correlation between circulating and plasma membrane cholesterol levels in T cells and, consequently, the amplitude of P2X7-induced cellular responses. Interestingly, the inhibition of P2X7 induced by plasma membrane cholesterol loading showed different profiles depending on the cellular function considered. Thus, the levels of inhibition of pore formation obtained in the MBCD/Chol group were significantly higher than those obtained in the HFC diet groups maintained for 16 and even 24 weeks. By contrast, the inhibition of CD62L shedding reached a maximum at 16 weeks of the HFC diet and could not be increased following MBCD/Chol treatment. Moreover, CD4^+^ T-cell subsets, which present both higher sensitivity to eATP and lower plasma membrane cholesterol content than CD8^+^ T-cell subsets, appeared to be more highly affected by plasma membrane cholesterol enrichment. These results suggest the existence of a dose-dependent effect on the cholesterol inhibition of P2X7, in particular, concerning pore formation, but not CD62L shedding, at least in our present experiments. As CD4^+^ and CD8^+^ T-cell subsets do not show the same initial characteristics in terms of plasma membrane cholesterol and lipid raft content, the direct and indirect effects of cholesterol could explain the differences observed in the amplitude of P2X7-induced cellular responses among these various subsets. Indeed, the modulation of protein function by plasma membrane cholesterol can either be direct, through the binding of cholesterol to the protein, or indirect, through changes in the fluidity and thickness of the plasma membrane. As we have previously shown, increases in plasma membrane cholesterol levels negatively affected calcium entry in response to eATP stimulation [29]. As the influx of calcium directly depends on changes in the P2X7 trimer conformation, these data suggest a direct influence of cholesterol on the sensitivity of P2X7 in agreement with [22]. By contrast, other P2X7-induced cellular responses, in particular PS externalization and CD62L shedding, appear to be more dependent on membrane composition, although they required an initial intracellular signal such as Ca^2+^ influx. Interestingly, CD62L shedding by ADAM 17 may be dependent on PS externalization [37]. Therefore, the impact of plasma membrane cholesterol on these cellular responses may be more highly associated with its influence on plasma membrane fluidity and protein mobility than on direct P2X7 sensitivity. Finally, ADAM 17 is constitutively located within cholesterol-enriched microdomains, but cholesterol depletion by MBCD increases ADAM 17-dependent shedding of membrane anchored proteins, which may be due to increased substrate accessibility. However, intracellular signals such as Ca^2+^ influx or PKC signaling are still required to initiate ADAM-dependent membrane protein shedding, even following cholesterol depletion. Altogether, our data contribute to explain why the modulation of plasma membrane cholesterol content does not similarly affect all P2X7-induced cellular responses in CD4^+^ and CD8^+^ T-cell subsets.

## 4. Materials and Methods

### 4.1. Reagents

ATP^4-^ (adenosine-5′-triphosphate), P2X7 receptor antagonist A438079, methyl-β-cyclodextrin (MBCD), FITC-conjugated cholera toxin B subunit (CTB), filipin III from Streptomyces filipinensis, cholesterol (≥99%), and bovine serum albumin (BSA) were all purchased from Sigma Aldrich (St. Louis, MO, USA). The YO-PRO-3 fluorescent probe (Molecular Probes) was purchased from Thermo Fisher Scientific (Les Ulis, France).

### 4.2. Mice

Wildtype C57BL/6J (B6) mice were obtained from The Jackson Laboratory (Bar Harbor, ME, USA). Animal protocols were approved by local and national ethics committees for research (agreement number: #20833). All mouse strains were maintained in our animal facilities (Plateforme Animex, Animalerie de l’Institut Paris-Sud d’Innovation thérapeutique).

### 4.3. Diet

Mice were either fed a normal diet (ND) or a high fat diet (34% lard, 8.3% saturated fatty acids, 4.5% mono-unsaturated fatty acid and 5.8% poly-unsaturated fatty acids) supplemented with 1% cholesterol (HFC) (Ssniff, Soest, Germany) [38]. All diets were supplied ad libitum for 16 to 24 weeks. The weight of the mice was monitored every week.

### 4.4. Metabolic Assay

Glucose sensitivity was assessed by an oral glucose tolerance test (OGTT). The glucose load (2 g/kg) was given by gavage after 6 h of fasting and blood samples were harvested at 0, 15, 30, 60, 90, and 120 min after gavage. Serum glucose concentrations were determined using the Accu-Chek Performa test (Roche, Basel, Switzerland) and the area under the glucose–time curve was calculated.

### 4.5. Serological Parameters

Circulating cholesterol levels were quantified in B6 mice serum using a AU400 chemistry analyzer (Olympus AU400, Biochemistry laboratory facility, UMR1149 INSERM-Université Paris Diderot, Paris, France).

### 4.6. Plasma Membrane Cholesterol Modulation

Plasma membrane cholesterol content was increased ex vivo by treating spleen cells from B6 mice with MBCD/Chol complexes at a 12:1 molar ratio in RPMI 1640 medium for 2 h at 37 °C. MBCD/Chol complexes were subsequently removed after extensively washing the cells with ice-cold RPMI 1640 medium. Plasma membrane cholesterol content was decreased by treating B6 spleen cells with 5 mM MBCD in RPMI 1640 medium for 20 min at 37 °C and then removed by washing cells extensively with ice-cold RPMI 1640 medium.

### 4.7. T Lymphocyte Phenotyping

Splenocyte From B6 mice fed a normal or high-fat diet were phenotyped by flow cytometry using fluorescent-conjugated monoclonal antibodies (mAbs) directed against the cell surface markers CD90.2/Thy1.2 (clone 30-H12), CD45RABC/B220 (clone RA3-6B2), CD4 (clone GK1.5), CD8α (clone 53-6.7), CD45RB (clone C363.16A), CD44 (clone IM7), CD62L (clone MEL-14), and P2X7 (clone 1F11) (all from eBioscience, San Diego, CA, USA). Cell membrane cholesterol was stained with filipin III. Binding of FITC-labeled CTB was used to measure ganglioside GM1 plasma membrane expression. Fluorescent-conjugated rat or mouse IgG1, IgG2a, or IgG2b, Armenian hamster IgG mAbs, or rabbit IgG polyclonal serum were used as isotype controls (eBioscience). Anti-mouse Fcγ receptor mAb (clone 93, eBioscience) were used to avoid non-specific antibody binding. At least 20,000 events were analyzed for each sample. Cell debris, dead cells, and doublets were gated out using the physical characteristics of cell-like granularity (side scatter, SSC) and size (forward scatter, FSC). Data acquisition was performed at the Flow Cytometry Core Facility at IPSIT (Clamart, France). Flow cytometry data were analyzed using FlowJo v10.8.1 (BD, Ashland, OR, USA) software.

### 4.8. ATP-Mediated Cellular Function Assays

Assays for P2X7-induced CD62L shedding, phosphatidylserine (PS) externalization, and pore formation were performed as previously described [16,17,18,39]. Briefly, splenocytes (10^6^ cells/mL) suspended in RPMI 1640 culture medium were stimulated with 500 µM eATP in a humidified 5% CO_2_ atmosphere at 37 °C for 30 min. Then, cells suspensions were washed and labelled for 30 min on ice with phenotype-specific fluorescent mAbs and fluorescent-conjugated anti-CD62L mAb, and Annexin V fluorescent probe to assess CD62L shedding and PS externalization. To measure P2X7-mediated pore formation, eATP stimulation was performed in the presence of YO-PRO-3 fluorescent dye (Thermo Fisher Scientific). In certain experiments, cells were pre-treated with 10 µM of the competitive P2X7 antagonist A438079 for 20 min before treatment with eATP. Data acquisition was performed at the Flow Cytometry Core Facility at IPSIT (Clamart, France). Flow cytometry data were analyzed using FlowJo v10.8.1 (BD, Ashland, OR, USA) software.

### 4.9. Statistical Analysis

Data are reported as the mean ± SEM. Experimental and control groups were compared using unpaired two-tailed Student’s *t*-tests or ANOVA. Statistical significance is indicated as follows: * *p* ≤ 0.05; ** *p* ≤ 0.01; *** *p* ≤ 0.001, **** *p* ≤ 0.0001, ns = not significant.

## Data Availability

Not applicable.
